# Myocardial Infarction Due to an Anomalous Origin of the Left Coronary Artery with Unique Aggravating Features

**DOI:** 10.5935/abc.20180217

**Published:** 2019-01

**Authors:** Jorge Alberto Silva Estrada, Alejandra Domínguez Camacho, Lorenzo Reyes de la Cruz, Jesus Reyna Figueroa

**Affiliations:** Department of Pediatrics - Hospital Central Sur de Alta Especialidad, PEMEX, Mexico City - Mexico

**Keywords:** Heart Defects Congenital, Myocardial Infarction, Echocardiography/methods, Magnetic Resonance Spectroscopy/methods, Myocardial Revascularization

## Introduction

Anomalous left coronary artery arising at the right sinus of Valsalva is a relatively rare congenital cardiac anomaly that can cause myocardial ischemia.^1^ It can follow one out of five aberrant courses: interarterial, subpulmonic, pre-pulmonic, retroaortic, or retrocardiac.^2^

We present the case of a young patient with an anomalous left coronary artery arising at the right sinus of Valsalva with severe stenosis and hypoplasia throughout the interarterial segment, which hampered corrective surgery. The timely detection and adequate treatment of this specific anomaly gains relevance because of its association with increased risk of sudden cardiac death. Although the true prevalence of interarterial anomalous left coronary artery is unknown, owing to the lack of population-wide screening studies and at times asymptomatic course, its frequency has been reported at 0.03%.^3^ Imaging techniques allow the characterization of the coronary anomalous origin, course, morphology and surrounding structures. Transthoracic echocardiography, magnetic resonance angiography, and computed tomography (CT) angiography are first line noninvasive tests available while invasive tests such as coronary angiography and intravascular ultrasound are second line diagnostic alternatives. Treatment approaches are still controversial and election of the optimal surgical procedure, whenever applicable, must be an individualized and patient-centered decision.

## Case report

**Figure 1:**
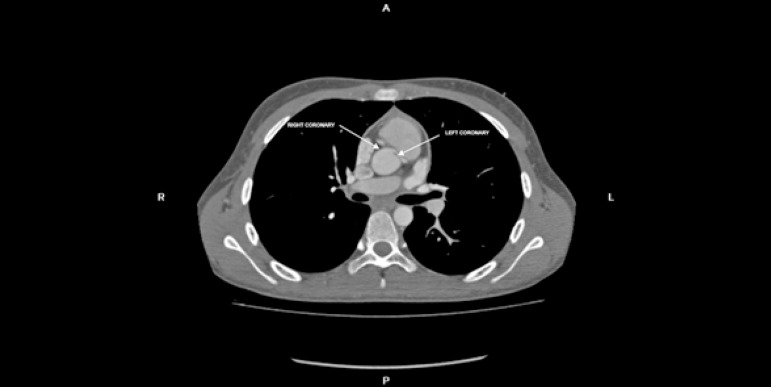
CT angiography shows anomalous origin of the left coronary artery arising at the right sinus of Valsalva from a separate ostium.

**Figure 2:**
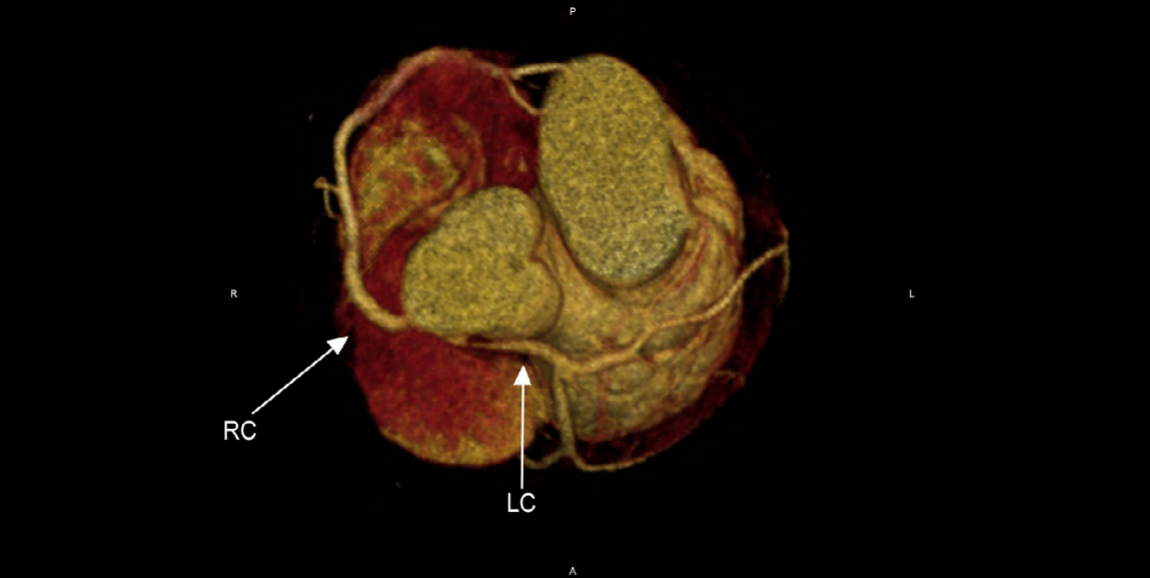
Three-dimensional reconstruction shows high-risk coronary anatomy: acute take-off angle, no intramural interarterial segment, long stenotic-hypoplastic segment (11 mm). RC: right coronary artery; LC: left coronary artery.

A fourteen-year-old otherwise healthy boy with no family history of disease presented with severe chest pain while he had been jogging for 5 minutes. The pain lasted for 2 hours and was followed by generalized weakness, dyspnea and confusional state. He was initially treated on a secondary care local clinic in which a baseline electrocardiogram reported ST segment depression in all precordial leads and serum Troponin I taken within 24 hours of symptom onset reached > 30 ng/mL (reference level of fluorescence immunoassay 0-0.4 ng/mL). The patient developed pulmonary edema and spent 7 days in the intensive care unit. After stabilization, he was referred to our tertiary care hospital. On hospital admission, he was hemodynamically stable, cardiac and pulmonary examination were normal. Plain chest x-ray was normal and the electrocardiogram showed sinus rhythm with ST segment depression and repolarization abnormalities in precordial leads V1 to V3. Complete blood count reported leukocytosis with neutrophilia; lipid profile and the toxicologic screening, including cocaine, came back normal. A transthoracic echocardiogram was performed which revealed a hypokinetic anteroseptal wall with normal systolic and diastolic function; no report of coronary anomalies was documented in the first place. Polymerase chain reaction tests for various viruses (Coxsackie type A and B, Parvovirus, Ebstein Barr, Cytomegalovirus, Poliovirus, Echovirus and Herpes Simplex 1,2,6,7 and 8) on peripheral blood samples were negative. He was pharmacologically managed with aspirin, atenolol and ivabradine. A rest perfusion magnetic resonance imaging detected an anterior, anteroseptal and lateral nontransmural myocardial infarction with systolic left ventricular dysfunction (ejection fraction of 45%) alongside an anomalous origin of the left coronary artery arising at the right sinus of Valsalva with an interarterial stenotic tract. A CT angiography demonstrated a left coronary artery arising at the right sinus of Valsalva from a separate ostium with an acute take-off angle and proximal oval-like narrowing with an extension of 11 mm running throughout the interarterial segment (Figures 1 and 2). Coronary translocation was discarded because the proximal interarterial segment was very stenotic and hypoplastic. Translocation was technically difficult and would not have restored normal coronary flow. Instead, through median sternotomy, cardiovascular surgeons performed revascularization of the anterior descending coronary artery with an internal mammary artery graft. Seven days after surgery he was discharged. The patient underwent treadmill stress testing according to the Bruce protocol and accomplished 9 sessions achieving a work level of 10.2 METS with adequate tolerance. He has been followed up in the cardiology outpatient clinic. Up to 18 months after surgery he is reported asymptomatic with normal electrocardiograms and echocardiographic evidence of normal systo-diastolic function. The cardiology team decided to restrict any strenuous physical activity.

## Discussion

We deem important the presentation and discussion of this case considering its unique high-risk features, particular evolution and nonstandard surgical approach with good clinical outcome.

Structural heart diseases are among the causes of sudden cardiac death in young patients.^4^^,^^5^ Clinicians must bear in mind that anomalous origin of the left coronary artery is a differential diagnosis in every previously healthy young patient with acute onset chest pain and evidence of acute myocardial ischemia. Transthoracic echocardiography can be the ideal diagnostic test in low-income settings; however, it must be noted that its accuracy is limited^6^ and a specific evaluation may obtain better results in the diagnosis. The detailed description of the anomaly should always be sought considering that there are three anatomical features that have been linked to a worse prognosis: intramural course, slit-like coronary ostium, and acute take-off angle of the anomalous coronary.^7^ In this case, apart from an acute take-off angle, a stenotic and hypoplastic course added to the disease burden. Given the aggravating coronary features encountered in this patient, aborted cardiac arrest or even sudden cardiac death could have been an expected outcome. Furthermore, the surgical approach could not tackle the coronary anomaly. Regardless of these apparently adverse factors, the patient fully recovered and reports asymptomatic with no evidence of cardiac lesion at more than one year follow up, which gives light to the fact that there must be other factors, such as vasoreactive ability and early collateral circulation, that can influence the course of this disease.

Corrective surgery, such as coronary translocation, must be offered to symptomatic patients with this coronary anomaly and high-risk features.^7^ Although the safety of corrective surgery has been demonstrated,^8^^,^^9^ its efficacy in the prevention of sudden cardiac death in the long term remains to be proven with further prospective studies. Besides, whenever we encounter aggravating features that make corrective surgery a difficult approach, coronary artery bypass grafting poses an alternative without undermining patient’s short- and long-term prognosis. Comparing these surgical approaches in cohort studies should be advocated.

The poorly understood physiopathology and natural history of this coronary anomaly hinder the development of risk stratification strategies and causes controversies in management algorithms. The presence of knowledge gaps regarding true worldwide prevalence, specific mechanisms of myocardial ischemia and optimal surgical options call for ongoing research to improve evidence-based decision making.
